# Novel Ti6Al4V Surface Treatment for Subperiosteal Dental Implants: Evaluation of Osteoblast-like Cell Proliferation and Osteogenic Response

**DOI:** 10.3390/ma18061234

**Published:** 2025-03-11

**Authors:** Roberto Campagna, Valentina Schiavoni, Loredana Rao, Fabrizio Bambini, Andrea Frontini, Francesco Sampalmieri, Eleonora Salvolini, Lucia Memé

**Affiliations:** 1 Department of Clinical Sciences, Polytechnic University of Marche, 60121 Ancona, Italy; v.schiavoni@pm.univpm.it (V.S.); f.sampalmieri@univpm.it (F.S.); e.salvolini@univpm.it (E.S.); 2 Department of Life and Environmental Sciences, Polytechnic University of Marche, 60121 Ancona, Italy; s1106668@pm.univpm.it (L.R.); a.frontini@univpm.it (A.F.); 3 Department of Life Sciences, Health and Health Professions, Link Campus University Città di Castello (Pg), 06012 Città di Castello, Italy; l.meme@unilink.it

**Keywords:** MG-63, Ti6Al4V, osteogenesis

## Abstract

Nowadays, custom-made subperiosteal implants are emerging as a solution in all those cases where there is lack of healthy bone tissue to support endosseous implants. The development of innovative techniques has allowed the production of grids that precisely match the patient’s anatomy. Elucidating the impact of laser-melted Ti6Al4V grids on both hard and soft tissues with which they come into contact is, therefore, mandatory. In this study, we analyzed the effects of five different surface treatments on a human osteoblast-like cell line (MG-63). In particular, the cell proliferation and osteogenic response were evaluated. Taken together, our data demonstrate that in our in vitro setting, the new surface treatment developed by Al Ti color could enhance osteogenesis and improve the stabilization of the implant to the residual bone by stimulating the best osteogenic response in MG-63 cells. Although further studies are required to validate our data in an in vivo model, our results provide the basis for future advances in implantology for the long-term maintenance of osseointegration.

## 1. Introduction

Osseointegrated implantology represents a common therapeutic choice supported by an extremely large body of literature, starting from the studies published by Branemark et al. and ending with those that keep being published [1]. The short and long-term success of dental implants relies on the presence of bone volumes capable of supporting osseointegration and of soft tissues able to maintain the biological seal, as underlined by many authors [2]. Bone quality and quantity, therefore, represent key variables for the correct planning of implant therapies [3,4,5,6]. The clinical scenarios in which there are no adequate bone volumes have been classified by Linkolm and Zarb [7], and the indications for choosing the proper regenerative therapy in the face of different types of atrophy have been recently published [8].

The study of methods for achieving adequate volumes of bone tissue in both quantitative and qualitative terms has thus become fundamental to set up a proper treatment plan, to provide recommendations on the time required to obtain the expected results, and to identify the risks associated with such maneuvers. A number of different techniques, among which is guided bone regeneration (GBR), have been recommended over the years, with a varying degree of clinical success [9]. The scientific discussion focuses on the choice of the biomaterial to be used and on the type of membranes that should be able to not only slow down the fastest cell strains but also maintain the barrier effect over time [10,11]. Many published studies concern the in vitro investigation of bioactive materials [12], as well as the adoption of static magnetic techniques [13,14], the type of membranes to be used, and the kind of technique to be implemented.

The long-term positive outcomes of all published techniques, especially the more complex ones, are influenced by the individual skills of the oral surgeon [15], the biological response of the subject, and not least, by the lifestyle adopted by the patient both during and after healing [16]. For these reasons, in large atrophies, regenerative techniques are often not accepted by the patient, who ultimately accepts a removable prosthesis, provided that this prosthesis is still able to be stable during chewing. In the past, and in particular in the 1990s [17], techniques for the production of subperiosteal implants to be used in patients with severe bone atrophy of the jaws or bone defects were implemented [18]. Unlike endosseous implants, which are embedded deep within the bone, subperiosteal implants are placed underneath the periosteum, resting on the top of the maxillary or mandibular bone without the need to drill it. Therefore, they are typically used in all those cases where there is not enough healthy bone tissue to support an endosseous implant [19]. The technique included two surgical interventions: during the first surgical session, the area to be treated was opened and skeletonized in order to take an alginate impression of the residual bone and then closed with sutures. This impression was used to realize a plaster model, on which a wax structure was designed. This structure was then melted and replaced by a cobalt–chrome one, carrying abutments to which the custom-made prostheses were later anchored. The metal structure was inserted below the periosteum and stabilized to the residual bone during the second stage of surgery. The numerous and frequent complications described in the literature led to the decline in use of this technique [20].

Through the advent of Cone Beam Computed Tomography (CBCT), a deeper knowledge of the materials and the adoption of innovative techniques have allowed to overcome the limits of the previous technique and enhanced the advantages of subperiosteal implants. In particular, EagleGrid (Bergamo, Italy) has patented a preparation system for the new generation of subperiosteal implants, publishing the results obtained in several scientific works; specifically, the preparation requires a sequence of several steps, including the patient’s scan with known reference points and the design of the subperiosteal grid with a dedicated software that allows to accurately assess both the anchorages and the physical resistance of the bone structures to them. Thanks to this innovative and controlled procedure, it is, therefore, possible to obtain a subperiosteal grid that precisely fits to the patient’s anatomy and is free from mechanical tensions [21]. The grid obtained with the laser melting technique has one surface that faces the bone structure, while the other faces the periosteum. The in vitro response of the bone to different surfaces, in particular, those obtained with laser melting, has been previously investigated [22,23]. Moreover, another research group [24] has published a work regarding the response of the periosteum to various surfaces, including those obtained by means of the laser melting technique used to produce the EagleGrid grids. In order to further improve the hard and soft tissue response to grids manufactured with laser melting, several surface treatments have been developed so far. In this context, the aim of our study was to evaluate the in vitro response of osteoblasts cultured on five Ti6Al4V discs subject to different surface treatments, among which is a novel treatment specifically intended for subperiosteal dental implants.

## 2. Materials and Methods

### 2.1. Experimental Design

MG-63 cells were seeded directly onto the surface of the discs made of grade 5 Ti6Al4V, which was 10 mm in diameter. The discs utilized in this study were manufactured by New Ancorvis S.r.l. (Bargellino di Calderara di Reno, Italy) and subject to various superficial treatments, which were performed by Al Ti Color S.r.l (Piazzola sul Brenta, Italy).

Specifically, disc no. 1 (ctrl) did not undergo any treatment, disc no. 2 was polished via electroerosion (EE), and disc no. 3 was subject to etching and sandblasting (ES). In addition, the surface of disc no. 4 was a novel superficial treatment designed by Al Ti color (ATcs), and disc no. 5 underwent color anodizing (CA). The investigators were only made aware of the surface treatment at the end of the study.

### 2.2. Scanning Electron Microscopy (SEM) Analysis

Scanning electron microscopy (SEM) observations were carried out to investigate the surface morphology of the different discs. Briefly, the samples intended for SEM analysis were mounted on aluminum stubs, gold-sputtered by Emitech K550 sputter coater (Quorum Technologies, Laughton, UK), and observed with a Zeiss SUPRA 40 SEM (Carl Zeiss, Oberkochen, Germany).

### 2.3. Cell Cultures

The MG-63 human osteosarcoma cell line, an established osteoblast-like cell model, was obtained from American Type Culture Collection (ATCC, Manassas, VA, USA). The cells were cultured using High Glucose Dulbecco’s Modified Eagle’s Medium (DMEM High Glucose, Euroclone, Pero, Italy), supplemented with 10% fetal bovine serum (FBS) and 50 µg/mL gentamicin (Euroclone, Italy). The cultures were kept at 37 °C in a humidified incubator with 5% CO_2_.

### 2.4. Cell Proliferation Assay

Cell proliferation was evaluated using a colorimetric assay that quantified the conversion of 3-(4,5-dimethylthiazol-2-yl)-2,5-diphenyl tetrazolium bromide (MTT, Merck Life Science S.r.l., Milano, Italy) into insoluble formazan, facilitated by the dehydrogenase enzymes of mitochondria in viable cells, as described in previous studies [25]. In brief, the proliferation of MG-63 cells seeded on differential surface-treated discs was assessed at several timepoints (0, 24, 48, and 72 h), while untreated surfaces were used as controls. Then, 3 × 10^4^ cells were cultured onto titanium discs in 24-well plates and let to adhere for 5 h at 37 °C. After this, the medium was removed and replaced with 700 µL of a fresh and complete one. Cell proliferation was evaluated at each time point by quantifying the conversion of tetrazolium salt MTT into formazan crystals. Specifically, 700 µL of fresh medium deprived of FBS and containing MTT reagent (5 mg/mL in PBS) was placed in all wells. After a 2 h incubation at 37 °C, the solution was discarded, and the formazan crystals were dissolved through the addition of 200 µL of 2-propanol (Merck, Milano, Italy) to each well. The resulting solution was transferred to a 96-well plate for absorbance measurement. The product resulted from the reaction was measured by evaluating absorbance at 540 nm through a microplate reader (Multiskan GO, Thermo Fisher Scientific Inc., Waltham, MA, USA). Results are presented as a percentage relative to the control (which is set at 100%, relative to the absorbance value of each sample at time 0) and expressed as the mean ± standard deviation from three independent experiments, each performed in triplicate.

### 2.5. Fluorescence Microscopy

Fluorescence microscopy was used to evaluate adhesion of cells to discs. As previously described, 3 × 10^4^ MG63 cells were cultured on titanium discs and after 72 h of incubation, the medium was discarded, and cells were fixed with 4% formaldehyde for 15 min at room temperature. Samples were stored at 4 °C in 700 µL of 1X PBS for subsequent analysis.

Alexa Fluor Plus 647 Phalloidin (ThermoFisher Scientific) was used for staining actin filaments. First, phalloidin was diluted 400 times in 1X PBS, and 700 µL of work solution was added to each well in which the discs were positioned. The samples were incubated for 1 h with agitation and then washed three times with 1X PBS. Subsequently, 50 µL of ProLong Glass Antifade Mountant with NucBlue (Invitrogen by Thermo Fisher Scientific) was positioned on the slide and the discs were placed such that the surface with the attached cells was in contact with the mountant. Following a 15 min incubation at room temperature, slides were examined using a NIKON AIR confocal fluorescence inverted microscope (Nikon Corporation, Tokyo, Japan) with a 20X objective. Images were captured using the NIS-Element imaging and analysis software (version 5.21.00; Nikon).

### 2.6. RNA Extraction and Reverse Transcription

RNA extraction and reverse transcription were performed after homogenizing MG63 cell pellets. Total RNA was isolated using SV Total RNA Isolation System (Promega, Madison, WI, USA), according to instructions of the manufacturer. RNA purity and concentration were assessed via nanodrop. Then, 1 µg of total RNA was reverse transcribed by using random primers and M-MLV Reverse Transcriptase (Promega, USA) at 37 °C for 60 min, and the cDNA obtained was stored at −20 °C up to the point of analysis.

### 2.7. Real-Time PCR

Human osteogenesis PCR array SBHS-026ZD, provided by Qiagen (Germantown, MD, USA), was utilized to assess the expression of 84 genes involved in osteogenic differentiation at 72 h. cDNA was combined with the QuantiNova™ SYBR^®^ Green PCR kit (Qiagen), and 20 μL aliquots were placed into each well of the PCR array. The amplification was performed using the CFX96 Real-Time PCR Detection System (Bio-Rad Laboratories, Hercules, CA, USA) with the following conditions: an initial cycle of 2 min at 95 °C, followed by 40 cycles of 5 s at 95 °C and 10 s at 60 °C. Data analysis was conducted using the ΔΔCt method as described in previous studies. Genes with Ct values ≥ 35 were considered undetectable (negative call) and assigned a value of 35. GAPDH gene served as the housekeeping gene for calculating ΔCt values of the target genes. Fluorescence emitted by the intercalating dye, which binds to double-stranded DNA, was detected to track the amplification of PCR products. Fold changes in gene expression were calculated using the 2^−ΔΔCt^ method, where ΔCt = Ct (Gene of interest) − Ct (GAPDH), and Δ(ΔCt) = −ΔCt (EE disc; ES disc; ATcs disc; CA disc) − ΔCt (CTRL disc). Ki-67 gene expression evaluation was performed as previously described [26]. Briefly, the following primers were used: for Ki-67 (forward) 5′-GACATC CGT ATC CAG CTTCC-3′ and (reverse) 5′-CCGTAC AGG CTC ATC AATAAC-3′, (forward) 5′-TCC TTC CTG GGC A TGG AGT -3′ and (reverse) 5′-AGC ACT GTG TTG GCG TAC AG-3′ for β-actin, the latter was used as a reference gene. SsoFast EvaGreen Supermix and CFX96 Real-Time PCR Detection System (Bio-Rad Laboratories, CA, USA) were utilized to run samples in duplicate for 40 cycles at 95 °C for 30 s and 58 °C for 30 s. Fold changes in the relative gene expression were estimated via the 2^−ΔΔCt^ method.

### 2.8. Statistical Analysis

The results were analyzed using GraphPad Prism 8.4.2 (679) software (GraphPad Software Inc., San Diego, CA, USA). Group differences were evaluated using a one-way ANOVA test, and a *p*-value < 0.05 was considered statistically significant.

## 3. Results

### 3.1. Scanning Electron Microscopy (SEM) Analysis

**Figure 1:**
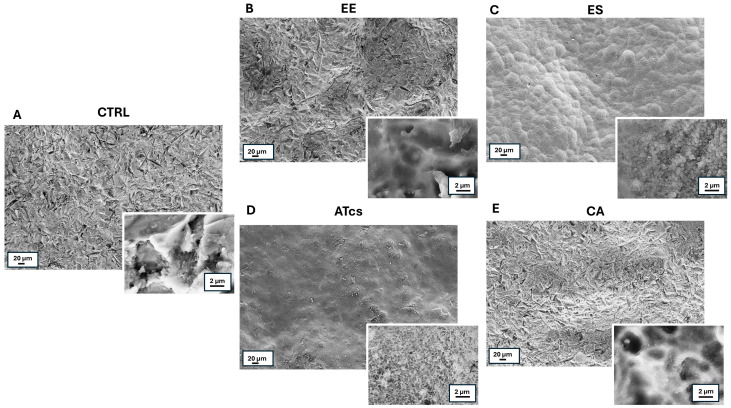
Low- and high-magnification (insets) SEM images of discs subject to different surface treatments. (**A**) CTRL disc; (**B**) EE disc; (**C**) ES disc. 3; (**D**) ATcs disc; (**E**) CA disc.

SEM observations of discs showed different surface morphology (Figure 1). In particular, the CTRL disc, which served as the control, exhibited a rough surface (Figure 1A). The morphological analysis of the EE disc revealed, especially at a higher magnification, the presence of superficial depressions of different sizes and depths (Figure 1B), while the combination of sandblasting and acid etching, to which the ES disc was subject, resulted in the creation of micro roughness over the rounded reliefs that characterize the sample surface (Figure 1C). As concerns the ATcs disc, at low magnification, its surface appearance is similar to that of the ES disc, while at a higher magnification, it showed the presence of superficial microporosities (Figure 1D). Finally, the CA disc exhibited an irregular surface, characterized, at a higher magnification, by circular as well as longitudinal depressions of variable size and depth (Figure 1E).

### 3.2. Evaluation of MG-63 Proliferation

**Figure 2:**
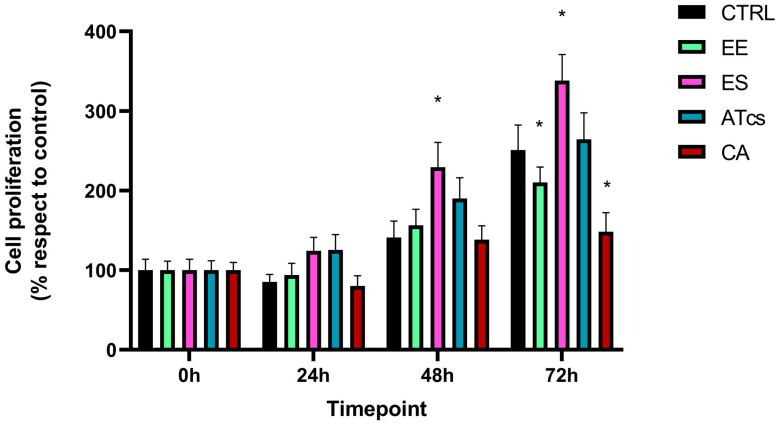
The cell proliferation of MG-63 cells evaluated via MTT assay at different timepoints (0, 24, 48, and 72 h). Values are expressed as mean ± standard deviation; * *p* < 0.05.

**Figure 3:**
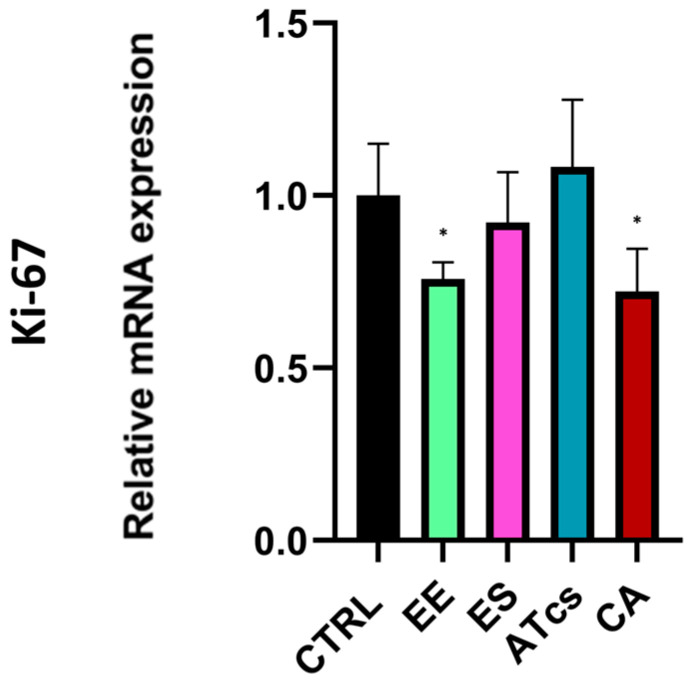
Ki-67 expression levels evaluated by Real-Time PCR at 72 h. Values are expressed as mean ± standard deviation; * *p* < 0.05.

The proliferative capacity of MG-63 cells, which were seeded and cultured on the surface of the different discs utilized in this study, was assessed via the MTT assay performed at different timepoints (Figure 2). The MTT assay revealed that the MG-63 cells seeded on the EE disc had proliferated significantly less at 72 h than the cells cultured on the control disc, with results of 250.92% ± 31.45 for MG-63 cultured on the CTRL disc and 210.27% ± 19.28 for cells grown on the EE disc. However, no statistically significant differences were detected at the earlier timepoints (Figure 2). Contact with the ES disc induced a significant increase in cell proliferation compared to the control group at 48 h and 72 h. In particular, the proliferation of MG-63 cells seeded on the ES disc at 24 h was 124.25% ± 16.90, while the control group exhibited a proliferation value of 85.24% ± 9.37; however, this value was not statistically significant. Instead, the proliferation of cells cultured on the control disc was 140.96% ± 20.6 at 48 h, but 229.45% ± 31.11 for cells grown on the ES disc; this difference continued to be evident at 72 h; at this time, the cells cultured on the control disc reached a value of 250.92% ± 31.45 while those seeded on the ES disc reached a value of 338.08% ± 32.89 (Figure 2). Regarding MG-63 cultured on the surface of the ATcs disc, no statistically significant differences in proliferation were detected between these cells and those cultured on the control disc (Figure 2). Among the discs tested, the CA disc negatively impacted cell proliferation at 72 h, with a proliferation value of 148.24% ± 24.10; in comparison, a value of 250.92% ± 31.45 was recorded for cells cultured on the control disc (Figure 2). Differences in cell proliferation at 72 h were further validated by assessing expression levels of Ki-67, a nuclear protein that is associated with cellular proliferation and ribosomal RNA transcription. In line with MTT assay results, the EE and CA discs showed lower Ki-67 expression levels compared to CTRL, while no statistically significant differences were observed for the ES and ATcs discs (Figure 3).

### 3.3. Fluorescence Microscopy

**Figure 4:**
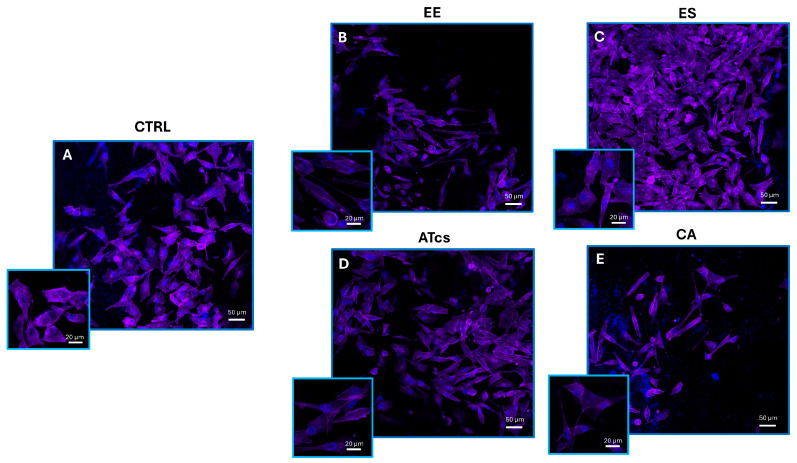
Representative confocal microscopy images of viable MG-63 cells cultured for 72 h on (**A**) control disc, (**B**) EE disc, (**C**) ES disc, (**D**) ATcs disc, and (**E**) CA disc. ProLong Glass Antifade Mountant with NucBlue was used for nuclei staining, while phalloidin was used to stain actin filaments. High-magnification pictures (20 µm scale bar) and inset with lower magnification pictures (50 µm scale bar).

The ability of MG-63 cells to colonize the different disc surfaces was investigated via fluorescence microscopy. Indeed, 72 h after seeding, viable cells were fixed and both the nucleus and cytoplasm were stained utilizing ProLong Glass Antifade Mountant with NucBlue and Alexa Fluor Plus 647 Phalloidin. For each sample, high (20 µm scale bar) and lower (50 µm scale bar) magnification pictures were captured (Figure 4). In accordance with the results reported in the previous paragraph, fluorescence microscopy showed that a larger number of MG-63 cells were seeded on the ES disc (Figure 4C). There were no relevant differences in cell density between the MG-63 cells seeded on the ATcs disc (Figure 4D) and those seeded on the CTRL disc (Figure 4A). Moreover, in accordance with previous data, a lower cell density was observed for MG-63 cells cultured on the EE and CA discs (Figure 4B,E).

### 3.4. Gene Expression Profiling

**Figure 5:**
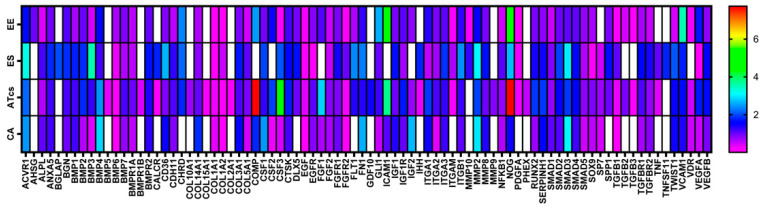
Heatmap of osteogenesis gene expression. Differential expression, at 72 h, of 84 genes involved in osteogenesis in MG-63 cells cultured on EE, ES, ATcs and CA discs compared to the control.

**Figure 6:**
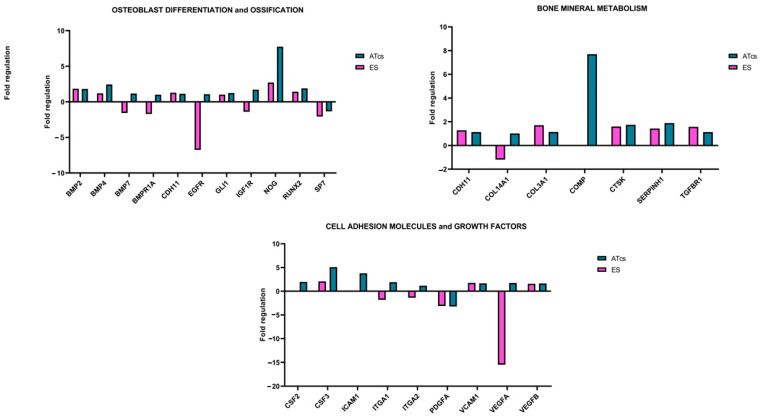
Fold regulation of the indicated genes after 72 h of cell culture on ES and ATcs discs 4 compared to the control.

The differential expression of 84 genes involved in osteogenesis was analyzed via an RT-PCR array (Figure 5). Several genes were found to be dysregulated in MG-63 cells cultured on discs characterized by different surface treatments; this is compared to cells cultured on the CTRL disc. The fold change expression of the 84 genes is reported in Figure 3, while Table 1 lists the exact value of the fold change in expression. Each surface treatment was able to dysregulate a number of the osteogenic genes analyzed, suggesting that surface treatment could play a key role in influencing gene expression. A two-fold increase in gene expression, in comparison to the control disc, was detected for the EE disc regarding genes CHRD, COMP, GLI1, ICAM1, NOG, and VCAM1; for the ES disc regarding genes ACVR1, BGLAP, BMP3, CD36, CHRD, CSF3, FLT1, FN1, ITGB1, MMP2, NOG, SMAD3, and TWIST1; for the ATcs disc regarding genes ACVR1, BMP4, COMP, CSF3, FGF1, ICAM1, and NOG; and for the CA disc regarding genes ACVR1, BMP4, CSF1, FN1, IGF2, MMP2, and SMAD3. Interestingly, surface treatment differentially suppressed the expression of the following genes: BGLAP, BMP5, BMPR1B, CALCR, COL10A1, COL15A1, COL2A1, GDF10, IHH, MMP9, PHEX, SP7, TNF, and TNFSF11 for the EE disc; AHSG, BMP5, BMPR1B, COL10A1, COL15A1, COL2A1, COMP, CSF2, FGF1, GDF10, ICAM1, IGF2, MMP9, PHEX, TGFB2, TGFB3, and VDR for the ES disc; AHSG, BGLAP, MMP10, and TNFSF11 for the ATcs disc; and AHSG, BGLAP, BMP5, BMPR1B, CALCR, CHRD, COL10A1, COL15A1, COL2A1, DLX5, FLT1, GDF10, IHH, ITGAM, MMP10, MMP9, PDGFA, PHEX, SPP1, TGFB2, TGFB3, TNF, and TNFSF11 for the CA disc. Figure 6 shows the quantification of the relative expression levels of genes involved in osteoblast differentiation, ossification, and bone mineral metabolism, as well as growth factors and cell adhesion molecules, after 72 h of culturing MG-63 cells on ES and ATcs discs.

## 4. Discussion

In this study, we analyzed the effects of five different surface treatments on a human-osteoblast-like cell line (MG-63) in order to evaluate the proliferation of cells and osteogenesis. In vitro experiments showed that the ES disc positively affected cell proliferation, starting from 48 h; meanwhile, the ATcs disc did not influence cell proliferation, leading to results that were comparable to the control disc. EE and CA discs negatively influenced the proliferation of cells at 72 h, with the data showing a significant reduction. Consistently, at 72h, the expression levels of Ki-67, a protein highly expressed during all active phases of the cell cycle, was found to be downregulated in cells cultured on the EE and CA discs, while for the ES and ATcs discs, its expression was comparable to CTRL. Nonetheless, Ki-67 expression levels do not always affect cell proliferation. Indeed, it has been reported that the main role of Ki-67, rather than promoting cell proliferation, is to contribute to organizing heterochromatin [27]. In order to investigate the impact of the surface treatment on the expression of genes involved in osteogenesis, an RT-PCR array was performed. The data obtained revealed the differential expression of genes involved in osteoblast differentiation, ossification, cell–cell adhesion, bone mineral metabolism, and genes encoding for growth factors. In particular, ES and ATcs discs showed the best expression of osteogenic genes in terms of the fold-change or the number of expressed genes. In particular, several osteoblast differentiation and ossification genes were differentially expressed, including BMP2, BMP4, BMP7, BMPR1A, CDH11, EGFR, GLI1, IGF1R, NOG, RUNX2, and SP7. Bone morphogenetic proteins (BMPs) are a family of proteins involved in the formation and maintenance of bone. It is known that BMPs induce the differentiation of mesenchymal precursor cells in osteoblasts, a differentiation process in which bone-specific genes, such as alkaline phosphatase and osteocalcin, are activated. BMP2 activates osteogenic genes and is known as the strongest osteoinductive factor; indeed, it triggers the differentiation of mesenchymal stem cells (MSCs) into osteoblasts and chondrocytes both in vivo and in vitro [28]. BMP4, which is essential in cartilage and bone development, is also involved in the mineralization of bone [29]. Moreover, BMP7 enhances the differentiation of osteoblasts and has been found to induce, in many cell types, the genetic markers of osteoblast differentiation; in addition, it induces the phosphorylation of SMAD1 and SMAD5 [30]. The BMPR1A signaling pathway stimulates the formation of osteoblastic bone and reduces bone resorption, leading to an increase in bone mass [31]. CDH11 and EGFR are involved in the differentiation of osteoblasts, while SP7 is a transcription factor that plays a key role in the differentiation of osteoblasts. GLI1 is known to be a marker of the skeletal progenitor pool, which contributes to the formation of bone during the healing of bone fractures [32]. IGF1R is a gene involved in the coordination of the chondrocyte, osteoclast, and endothelial response during the repair of bone fractures [33]. NOG is a BMP inhibitor that plays a crucial role in normal osteoblastogenesis and bone formation [34]. RUNX2 is considered an osteoblast master regulator; indeed, it plays a crucial role in osteogenesis and osteoblast maturation. RUNX2 cooperates with BMP-specific receptor-regulated Smad proteins and is an essential regulator of osteocalcin and alkaline phosphatase gene promoters [35]. It is important to notice that the RUNX2 gene is often analyzed in the context of surface-treated porous titanium implants [36]. In particular, Yavari et al. observed that alkali—acid—heat treatment can enhance the expression of RUNX2 compared to controls [24]. Shu et al. reported that the selective laser-melted implant subject to chemical oxidation displays higher RUNX2 levels than controls [37]. However, in these studies, the detected effect was delayed for several days with respect to our findings. Osteocalcin, osteopontin, bone sialoproteins, and many others are target genes of RUNX2. When RUNX2 is activated in pre-osteoblasts, the pre-osteoblasts undergo a multi-stage differentiation process that ultimately leads to matrix mineralization. This process is enhanced by the accumulation of osteocalcin in the organic matrix, which aids in the deposition of mineral substances. Osteocalcin is, in fact, the second most abundant protein in bone, following collagen. Although the proliferation of MG-63 cells was higher when cells were cultured on the ES disc compared to the ATcs disc, the analysis of gene expression revealed that the differentiation of osteoblasts was higher in cells cultured on the ATcs disc compared to those cultured on the CTRL disc and ES disc; this suggests that the surface treatment used for the ATcs disc could enhance osteogenesis, resulting in a better stabilization of the implant.

In accordance with the above-mentioned data, the ATcs disc was found to induce the expression of genes related to bone mineral metabolism, including CDH11, COL3A1, COMP, CTSK, SERPINH1, and TGFBR1. In addition, COL14A1 and COMP were expressed at very high levels. COL3A1 encodes for Type III collagen, a fibril-forming collagen, and is a major extracellular matrix component expressed by osteoblasts in mature bone; meanwhile, COL14A1 seems to play a crucial role in the formation of alveolar bone [38,39]. COMP, also known as cartilage oligomeric matrix protein, is an important extracellular matrix protein that plays a role in the functioning of musculoskeletal tissues and bone growth [40]. CTSK encodes for the protein cathepsin K, which plays an important role in bone turnover [41]. The SERPINH1 gene encodes for the protein HSP47 (heat shock protein 47), which acts as a molecular chaperone for type I procollagen, stabilizing the molecule in the rough endoplasmic reticulum and supporting its folding; this prevents the premature aggregation of its chains [42]. TGFBR1 plays a critical role in the development, homeostasis, and repair of bone. TGF-β signaling through TGFBR1 influences the differentiation of osteoblasts and promotes the maturation of these cells, thus contributing to bone formation. Moreover, TGF-β signaling stimulates osteoblasts to produce extracellular matrix proteins, such as collagen, and affects the balance between osteoblast and osteoclast activity. It is also involved in the regulation of bone density [43,44]. The ATcs disc was found to have the best expression pattern for bone mineral metabolism genes compared to the CTRL disc and ES disc; this suggests that the surface treatment used for the ATcs disc is able to stimulate the expression of bone mineral metabolism genes in vitro.

Finally, the PCR-array analysis showed the important expression of genes that encode for growth factors and cell adhesion molecules, including CSF2, CSF3, ICAM1, ITGA1, ITGA2, PDGFA, VCAM1, VEGFA, and VEGFB. Notably, CSF2 and CSF3 are colony-stimulating factors, which play a key role in bone homeostasis and are involved in the formation of osteoclasts and osteoclast-mediated bone resorption [45]. The increased gene expression of the adhesion molecules ICAM1 and VCAM1, which influence the proliferation of T lymphocytes in inflammatory settings and play a role in osteoblast–osteoclast interactions during osteoclastogenesis and the healing of bone fractures, further suggests that, in the context of bone physiology, the surface treatment used on ATcs disc may induce an osteoclast- and immunomodulatory bioresponse in osteoblasts, stimulating osseointegration. The expression of integrins ITGA1 and ITGA2 has been linked to surface roughness and has been reported to be a marker of the osteogenic differentiation of human mesenchymal stem cells on titanium surfaces [46]. Finally, VEGFA and VEGFB are angiogenic molecules that play a significant role in regulating blood vessel invasion, cartilage remodeling, and the ossification of newly secreted bone matrix [47]. Yavari et al. reported that the anodizing-heat treatment of surface-treated porous titanium enhances the VEGFA expression with a variable pattern, depending on the duration of cell-surface contact [24].

Taken together, our data demonstrate that in an in vitro setting, the surface treatment used for the ATcs disc could enhance osteogenesis and the integration of the implant. Although further studies are required to validate our data in an in vivo model, our results provide the basis for future advances in implantology.

## Figures and Tables

**Table 1:** Fold change of 84 genes at 72 h in MG-63 cells cultured on EE, ES, ATcs, and CA discs compared to the control (CTRL).

	Timepoint 72 h			
Gene	EE	ES	ATcs	CA
ACVR1	1.64	0.10	0.22	0.18
AHSG	1.00	Not detectable	Not detectable	Not detectable
ALPL	0.41	1.17	0.62	1.03
ANXA5	0.98	1.81	1.20	1.20
BGLAP	Not detectable	2.02	Not detectable	Not detectable
BGN	1.11	1.85	1.17	0.98
BMP1	0.69	1.76	1.19	1.20
BMP2	1.00	1.83	1.81	0.62
BMP3	0.46	3.57	1.28	0.41
BMP4	1.62	1.19	2.44	2.85
BMP5	Not detectable	Not detectable	0.36	Not detectable
BMP6	0.69	0.31	0.23	0.18
BMP7	0.89	0.64	1.16	0.78
BMPR1A	0.58	0.59	1.00	0.57
BMPR1B	Not detectable	Not detectable	0.42	Not detectable
BMPR2	1.02	1.68	1.39	1.29
CALCR	Not detectable	1.76	0.25	Not detectable
CD36	0.86	2.65	0.89	1.47
CDH11	0.58	1.28	1.13	0.86
CHRD	2.36	2.17	1.81	Not detectable
COL10A1	Not detectable	Not detectable	0.74	Not detectable
COL14A1	0.64	0.84	1.01	1.85
COL15A1	Not detectable	Not detectable	0.26	Not detectable
COL1A1	0.17	0.04	0.30	0.37
COL1A2	0.07	0.51	0.22	0.03
COL2A1	Not detectable	Not detectable	0.25	Not detectable
COL3A1	0.90	1.71	1.14	1.49
COL5A1	0.45	1.05	0.90	0.47
COMP	2.49	Not detectable	7.70	1.80
CSF1	0.95	1.97	1.27	2.46
CSF2	1.45	Not detectable	1.95	0.29
CSF3	0.12	2.07	5.08	0.44
CTSK	1.18	1.60	1.74	1.19
DLX5	0.92	1.67	0.99	Not detectable
EGF	0.29	0.26	0.40	0.17
EGFR	0.86	0.15	1.07	0.85
FGF1	1.11	Not detectable	2.84	0.66
FGF2	0.50	0.49	0.85	0.68
FGFR1	0.91	1.41	0.89	0.97
FGFR2	0.10	1.01	0.27	0.09
FLT1	0.66	2.38	1.34	Not detectable
FN1	1.28	2.30	1.33	2.61
GDF10	Not detectable	Not detectable	1.44	Not detectable
GLI1	2.60	1.00	1.23	0.51
ICAM1	4.44	Not detectable	3.77	1.09
IGF1	0.92	1.67	0.99	1.35
IGF1R	1.07	0.72	1.71	1.02
IGF2	0.82	Not detectable	0.76	2.67
IHH	Not detectable	1.78	0.33	Not detectable
ITGA1	1.33	0.55	1.91	1.44
ITGA2	0.93	0.72	1.15	0.37
ITGA3	1.06	1.86	1.26	1.22
ITGAM	0.14	0.67	0.26	Not detectable
ITGB1	1.28	2.42	1.24	1.08
MMP10	0.28	1.27	Not detectable	Not detectable
MMP2	1.51	3.07	1.95	2.95
MMP8	0.92	1.67	0.99	1.35
MMP9	Not detectable	Not detectable	0.86	Not detectable
NFKB1	0.46	1.09	0.78	0.62
NOG	4.76	2.70	7.74	1.45
PDGFA	0.19	0.32	0.31	Not detectable
PHEX	Not detectable	Not detectable	0.54	Not detectable
RUNX2	0.92	1.42	1.89	0.77
SERPINH1	0.82	1.43	1.89	1.58
SMAD1	0.50	0.88	0.78	1.06
SMAD2	0.98	1.96	1.52	1.12
SMAD3	1.04	3.00	1.90	3.08
SMAD4	1.05	1.62	1.55	1.22
SMAD5	0.79	0.64	0.83	0.50
SOX9	0.69	0.07	0.89	0.57
SP7	Not detectable	0.48	0.75	0.45
SPP1	0.92	1.67	0.99	Not detectable
TGFB1	0.02	1.05	0.34	0.01
TGFB2	0.45	Not detectable	0.93	Not detectable
TGFB3	0.23	Not detectable	0.11	Not detectable
TGFBR1	0.68	1.57	1.13	0.70
TGFBR2	0.52	1.39	0.79	0.86
TNF	Not detectable	1.02	0.38	Not detectable
TNFSF11	Not detectable	1.78	Not detectable	Not detectable
TWIST1	0.45	2.07	1.47	1.44
VCAM1	3.54	1.76	1.65	1.36
VDR	0.28	Not detectable	0.48	0.50
VEGFA	1.71	0.06	1.73	0.50
VEGFB	0.81	1.57	1.63	0.92

## Data Availability

The original contributions presented in this study are included in the article. Further inquiries can be directed to the corresponding authors.
